# Frequency and Prognostic Relevance of Volumetric MRI Changes in Contrast- and Non-Contrast-Enhancing Tumor Compartments between Surgery and Radiotherapy of IDHwt Glioblastoma

**DOI:** 10.3390/cancers15061745

**Published:** 2023-03-14

**Authors:** Nico Teske, Nina C. Teske, Maximilian Niyazi, Claus Belka, Niklas Thon, Joerg-Christian Tonn, Robert Forbrig, Philipp Karschnia

**Affiliations:** 1 Department of Neurosurgery, Munich University Hospital, LMU Munich, 81377 Munich, Germany; 2 German Cancer Consortium (DKTK), Partner Site Munich, 80336 Munich, Germany; 3 Department of Radiation Oncology, Munich University Hospital, LMU Munich, 81377 Munich, Germany; 4 Bavarian Center for Cancer Research (BZKF), 91054 Erlangen, Germany; 5 Institute of Neuroradiology, Munich University Hospital, LMU Munich, 81377 Munich, Germany

**Keywords:** glioblastoma, IDH-wildtype, surgery, radiotherapy, extent of resection, tumor progression, tumor residuals

## Abstract

**Simple Summary:**

The prognostic impact of early tumor regrowth in glioblastoma patients diagnosed according to WHO 2021 classification between surgery and postoperative adjuvant therapy remains unclear. In a retrospective cohort of 64 patients, we performed volumetric analyses of routine MRI obtained early after tumor resection and directly before initiation of radio(chemo-)therapy (time interval: 15.5 ± 1.9 days). About one third developed new contrast-enhancement after complete or incomplete resection due to progression of tumor remnants or breakdown of the blood–brain barrier in previously non-enhancing tumor compartments, but also related to postoperative ischemia and unspecific signals. Findings suggestive for tumor regrowth were not associated with unfavorable outcomes. Immediate postoperative MRI should, therefore, be used for prognostic stratification of the extent of resection, while additional imaging before the start of radiotherapy may be helpful for tumor volume and radiation target delineation.

**Abstract:**

In newly diagnosed IDH-wildtype glioblastoma, the frequency and prognostic relevance of tumor regrowth between resection and the initiation of adjuvant radiochemotherapy are unclear. In this retrospective single-center study we included 64 consecutive cases, for whom magnetic resonance imaging (MRI) was available for both the volumetric assessment of the extent of resection immediately after surgery as well as the volumetric target delineation before the initiation of adjuvant radiochemotherapy (time interval: 15.5 ± 1.9 days). Overall, a median new contrast-enhancement volume was seen in 21/64 individuals (33%, 1.5 ± 1.5 cm^3^), and new non-contrast lesion volume in 18/64 patients (28%, 5.0 ± 2.3 cm^3^). A multidisciplinary in-depth review revealed that new contrast-enhancement was either due to (I) the progression of contrast-enhancing tumor remnants in 6/21 patients or (II) distant contrast-enhancing foci or breakdown of the blood–brain barrier in previously non-contrast-enhancing tumor remnants in 5/21 patients, whereas it was unspecific or due to ischemia in 10/21 patients. For non-contrast-enhancing lesions, three of eighteen had progression of non-contrast-enhancing tumor remnants and fifteen of eighteen had unspecific changes or changes due to ischemia. There was no significant association between findings consistent with tumor regrowth and a less favorable outcome (overall survival: 14 vs. 19 months; *p* = 0.423). These findings support the rationale that analysis of the postsurgical remaining tumor-volume for prognostic stratification should be carried out on immediate postoperative MRI (<72 h), as unspecific changes are common. However, tumor regrowth including distant foci may occur in a subset of IDH-wildtype glioblastoma patients diagnosed per WHO 2021 classification. Thus, MRI imaging prior to radiotherapy should be obtained to adjust radiotherapy planning accordingly.

## 1. Introduction

Glioblastoma is the most frequent primary brain tumor in adults and identifies with aggressive growth [1]. Microsurgical resection represents the current standard of care when followed by adjuvant radiochemotherapy and consolidation chemotherapy [2]. The extent of surgical tumor resection is prognostic for survival [3]; however, recurrence inevitably occurs even when a complete tumor resection on postoperative magnet resonance imaging (MRI) has been achieved. In a subset of patients, changes in the contrast-enhancing (CE) or non-CE tumor portions might even be seen in the interval between the immediate postoperative imaging and imaging obtained prior to radiotherapy for the purpose of target delineation. Such changes have often been interpreted as tumor regrowth, and are therefore denoted by the term ‘rapid early progression’, but there are contradictory results on whether such findings are indeed associated with a less favorable outcome [4,5]. Consequently, the current guidelines on the treatment and management of adult patients with diffuse gliomas do not routinely recommend additional MRI acquisition before the initiation of postoperative radiotherapy [2,6].

The 2021 World Health Organization (WHO) classification of central nervous system (CNS) tumors prerequisites astrocytic gliomas to exhibit isocitrate dehydrogenase 1/2 (IDH)-wildtype status (together with classic histopathological hallmarks or other qualifying molecular features) for the diagnosis of ‘glioblastoma CNS WHO grade 4’ [7]; the category of ‘IDH-mutant glioblastomas’ has now been abandoned. Importantly, only two studies controlled for IDH status when assessing ‘rapid early progression’ [8,9], and Wee et al. [9] described a substantially higher incidence of tumor regrowth within the interval between surgery and the initiation of radiotherapy in IDH-wildtype than in IDH-mutant tumors. Accordingly, it remains unclear whether prior studies on the prognostic role of ‘rapid early progression’ might have been confounded by IDH status, as IDH-wildtype tumors identify with a more aggressive clinical course, but also by the heterogenous adjuvant treatment which was provided.

In the current manuscript, we present a cohort of 64 consecutive IDH-wildtype glioblastoma patients who were resected followed by irradiation, plus chemotherapy in most cases. Each patient had an immediate postoperative MRI as well as an MRI prior to the initiation of radiotherapy. We volumetrically assessed the frequency and extent of changes in CE and non-CE tumor, and a multidisciplinary in-depth imaging review served as the basis to distinguish true tumor progression from changes different in nature. Using this approach, we aimed to elaborate on the prognostic role of these early MRI changes in tumor morphology in the era of the 2021 WHO classification.

## 2. Materials and Methods

### 2.1. Study Population

We conducted a retrospective data search of our institutional database at the Department of Neurosurgery of the Ludwig-Maximilians-University in Munich, Germany. The study protocol and design were approved by the Institutional Review Board of the Ludwig-Maximilians-University in Munich, Germany (AZ:21-0996). The requirement to obtain informed patient consent was waived. Patients were included based on the following criteria: (1) tissue-based diagnosis of a glioblastoma, IDH-wildtype, CNS WHO grade 4 according to the 2021 WHO classification of CNS tumors [7]; (2) first-line treatment consisting of microsurgical tumor resection followed by radio(chemo-)therapy; (3) MRI data available for review after tumor resection and immediately before initiation of radiotherapy sufficient for volumetric analyses, including ceT1-weighted and T2-weighted sequences (Figure S1). Data were collected on demographic and clinical information, histopathologic and molecular diagnostics, imaging, treatment specifics, and outcome data. Diagnostic and treatment decisions were based on the recommendation of a multidisciplinary neuro-oncology tumor board and patient preference. For radiotherapy, target volume delineations were conducted in accordance with the ESTRO-ACROP guidelines, based on contrast-enhancing T1- and T2-weighted MRI abnormalities on imaging obtained less than two weeks prior to the initiation of irradiation [10].

### 2.2. Magnetic Resonance Imaging

Evaluation of MRI at first diagnosis, immediately after microsurgical tumor resection and prior to radio(chemo-)therapy, was performed in accordance with the RANO criteria [11]. Further follow-up surveillance scans were obtained per current guidelines every 3–6 months or following clinical deterioration [2]. All MRI scans included ceT1-weighted and T2(-FLAIR)-weighted sequences, and postoperative imaging was obtained <72 h after surgery whenever possible. For all patients, we routinely applied a macrocyclic gadolinium-based contrast agent (gadoteric acid, Dotagraf 0.5 mmol/mL; 0.2 mL/kg bodyweight; 0.1 mmol/kg; flow rate 1 mL/s). Volumetric image analyses were performed as previously described [3]. In short, tumor volumes were quantified by ceT1-weighted and FLAIR (or if not available, T2)-sequences on pre-/postoperative and pre-radiotherapy MRI scans using commercially available imaging software (BrainLab^®^ Elements; Munich, Germany). Raters carefully ensured that postoperative FLAIR/T2 abnormalities were not surgically induced edema or ischemia by reviewing pre-operative T1 and T2 imaging as well as postoperative DWI sequences. Preoperative and residual tumor volumes were recorded in absolute numbers (in cm^3^), and the extent of resection (EOR) was calculated. Patients were subsequently assigned their corresponding RANO category 1–3 for EOR in glioblastoma. For more detailed analyses, a multidisciplinary in-depth review of all MRI scans, including diffusion-weighted-sequences, was performed by neurosurgeons and neuroradiologists with experience in glioma-imaging to further evaluate volumetric changes in contrast- and non-contrast-enhancing tumors portions.

### 2.3. Neuropathological Diagnosis

Histopathological diagnosis according to the 2021 WHO classification was based on tissue obtained during microsurgical tumor resection [7].

Methylation-specific polymerase chain reaction and Sanger sequencing of the Cytosine-Guanine dinucleotide (CpG) sites 74–98 within the O6-methylguanine-DNA-methlytransferase (MGMT) promotor region were used to analyze MGMT promotor status, as previously described [12]. Isocitrate dehydrogenase 1/2 was assessed per pyrosequencing; telomerase reverse transcriptase promotor mutation (TERT) status was analyzed using Sanger sequencing [13,14]. In the case of extended molecular diagnostics, DNA methylation-based tumor diagnostics or next-generation sequencing were performed whenever deemed clinically necessary [15].

### 2.4. Statistical Analysis

Absolute numbers and percentages were calculated for categorical variables. If not indicated otherwise, mean ± standard error of the mean are used to describe numerical data, and range is given. Chi-squared test was used to analyze associations between two or more categorical variables. The D’Agostino–Pearson omnibus normality test was used to test for normal distribution and equal variance in continuous data. In case of parametric data, differences between two groups were determined by the use of the unpaired Student’s t test; differences between more than two groups were assessed by use of a one-way analysis of variance (ANOVA). For non-parametric data, Mann–Whitney U-test and Kruskal–Wallis test were used to analyze differences between two and multiple groups, respectively, and Wilcoxon matched-pairs signed rank test was used in paired data. Kaplan–Meier survival estimates and log-rank test were calculated for survival analyses. Overall survival (OS) was defined as the interval from diagnosis to death from any cause, and progression-free survival (PFS) was defined as the interval from diagnosis to progression after first-line therapy or death from any cause. Patients were followed until death or day of database closure (1 December 2022). Patients lost to follow-up were censored at day of last follow-up. Date of diagnosis was defined as date of tumor resection of biopsy. Statistical analyses were performed using Prism statistical software (Prism 9.4; GraphPad Software Inc., San Diego, CA, USA). The significance level was set at *p* ≤ 0.05.

## 3. Results

### 3.1. Patient Characteristics and Adjuvant Therapies

**Figure 1:**
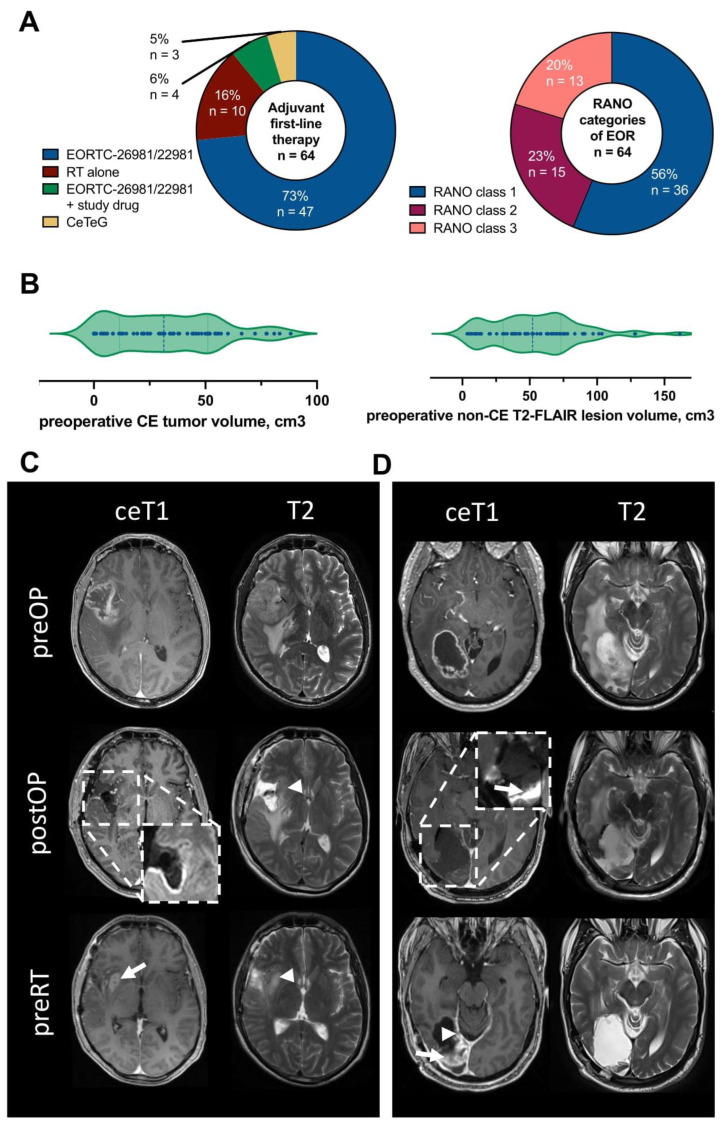
Baseline patient characteristics and volumetric analyses. (**A**) Distribution of administered first-line adjuvant therapies (left) and stratification of all patients according to the RANO *resect* classes for extent of resection (right) in patients with newly diagnosed glioblastoma CNS WHO grade 4 after undergoing microsurgical tumor resection (*n* = 64). (**B**) Preoperative CE tumor and non-CE T2-FLAIR lesion volume (in cm^3^) before microsurgical tumor resection (*n* = 64). (**C**,**D**) Contrast-enhancing T1- and T2-weighted MRI is shown for two patients displaying volumetric changes in CE and non-CE compartments due to (**C**) residual non-CE T2-FLAIR lesion progression (arrowheads) and breakdown of the blood–brain barrier leading to new CE tumor (arrow), and (**D**) postoperative ischemia (arrows in diffusion-weighted imaging inlay) and unspecific changes such as surgical scarring (arrowhead). Abbreviations: CE—contrast-enhancing; ceT1—contrast-enhancing T1-weighted MRI; CeTeG: RT/TMZ → TMZ/CCNU; CNS—central nervous system; EOR—extent of resection; EORTC 26981/22981: RT/TMZ → TMZ; preOP—preoperative MRI; postOP—immediate postoperative MRI; preRT—MRI prior to radio(chemo-)therapy; RANO—Response Assessment in Neuro-Oncology; RT—radiotherapy; non-CE—non-contrast-enhancing; T2—T2-weighted MRI.

A total of 663 glioblastoma patients consecutively treated at our Center of Neuro-Oncology between 2016 and 2022 were screened, and 64 patients matched the inclusion criteria (Figure S1). Mean age at diagnosis was 60.1 ± 1.3 years (range: 30–86), with a male-to-female ratio of 1:0.5 and preoperative Karnofsky Performance Status (KPS) 85% (range: 20–100%). Of note, one patient had a preoperative KPS of 20% as he presented with acute brain herniation and a decreased level of consciousness. MGMT promotor was methylated in 22/64 patients (34%), and TERT promotor mutation was present in 10/64 patients (16%). New postoperative deficits after microsurgical tumor resection were encountered in eight of sixty-four patients (13%), generally mild in nature. As such, median postoperative KPS did not differ from preoperative KPS (*p* = 0.210). Treatment strategies included standard-of-care radiochemotherapy per EORTC-26981/22981 protocol in 47/64 patients (73%), and radiotherapy in case of elderly patients with unmethylated MGMT promotor status (10/64 patients, 16%), respectively. In the remaining cases, radiochemotherapy was combined with lomustine according to CeTeG/NOA09-protocol or experimental drugs were added to the therapy regimen (Figure 1A; Table 1) [16].

### 3.2. MRI and Volumetric Analysis

Tumors were most frequently located (sub-)cortically (56/64 patients; 88%), followed by multifocal disease at first diagnosis in five of sixty-four patients (8%). Median preoperative (pre-op) CE tumor volume and non-CE T2-FLAIR lesion volume were 30.5 ± 3.1 cm^3^ (range: 0–88 cm^3^) and 53.9 ± 4.2 cm^3^ (range: 4–162 cm^3^; Figure 1B), respectively. In most cases, no residual tumor volume was detected on immediate postoperative (postOP) MRI with median postoperative CE tumor volume 0.0 ± 0.4 cm^3^ (range: 0–24 cm^3^) and median postoperative non-CE T2-FLAIR lesion volume 0.0 ± 1.6 cm^3^ (range: 0–64 cm^3^). In detail, residual tumor volumes were allocated to their respective RANO *resect* classes for EOR in glioblastoma [3]. Here, supramaximal CE resection (class 1) was achieved in 36/64 patients (56%), maximal CE resection (class 2) was possible in 15/64 patients (23%), and the remaining 13/64 patients (20%) underwent submaximal CE resection (class 3; Figure 1A). Of note, immediate post-op MRI < 72 h after surgery was available for 56/64 patients (88%).

The median time interval between early postoperative MRI and second MRI prior to the initiation of radio(chemo-)therapy (pre-RT) was 15.5 ± 1.9 days (range: 4–109 days). Of note, one patient receiving pre-RT MRI 109 days after post-op MRI represented an outlier due to prolonged neurological rehabilitation until a clinically stable condition allowed adjuvant therapy. In pre-RT analyses, median CE tumor and non-CE T2-FLAIR lesion volumes were 0.3 ± 0.9 (range: 0–56 cm^3^) and 1.3 ± 2.1 cm^3^ (range: 0–89 cm^3^). Median pre-op CE tumor volume significantly correlated with pre-RT CE tumor volume (* *p* = 0.017), but not with post-op CE tumor volume (*p* = 0.140). In the case of non-CE T2-FLAIR lesion, median pre-op volume correlated with both post-op and pre-RT volumes (* *p* = 0.023 and * *p* = 0.001).

Overall, 22/64 patients (34%) demonstrated positive volumetric changes between post-op and pre-RT MRI; no decreasing volumetric changes were observed. In these cases, multidisciplinary in-depth analysis was carried out to further characterize the observed changes. A median new contrast-enhancement volume of 1.5 ± 1.5 cm^3^ (range: 0.4–32.4 cm^3^) could be demonstrated in 21/64 patients (33%), and a new non-contrast lesion volume of 5.0 ± 2.3 cm^3^ (range: 0.5–35.3 cm^3^) in 18/64 patients (28%). Interestingly, a new contrast-enhancement volume was observed due to I) progression of CE tumor remnants in six of twenty-one patients (29%) and II) distant contrast-enhancing foci or breakdown of the blood–brain barrier (BBB) in previously non-CE T2-FLAIR lesion remnants in five of twenty-one patients (24%), whereas detailed analysis revealed unspecific changes or ischemia, due to respective changes in diffusion-weighted sequences, in the remaining 10/21 patients (48%; Figure 1C,D). A review of non-contrast lesion volumes revealed progression of non-CE remnants in only three of eighteen patients (17%), while the majority were unspecific or due to ischemia in 15/18 patients (83%). Moreover, ischemia could be demonstrated in 23/64 patients (36%); however, not all of them were assessable by volumetric analyses (i.e., faint linear contrast-enhancements and diffusion restrictions visible in the respective sequences; Figure 1D).

We also compared clinical, molecular, and imaging characteristics between patients demonstrating volumetric changes of tumor remnants (22/64 patients, 34%) and those who did not (42/64 patients, 66%; Table 1). Importantly, the median time to pre-RT MRI after post-op MRI did not significantly differ between both groups (16 ± 5.3 versus 15 ± 0.7 months; *p* = 0.350). Age, pre-/post-op KPS and MGMT promotor methylation did not differ between both groups (*p* = 0.123, *p* = 0.133, *p* = 0.394, *p* = 0.268); however, the TERT promotor mutation was more frequently encountered in patients with volumetric changes of tumor remnants (100% versus 76%, *p* = * 0.012). Interestingly, pre- and post-op non-CE T2-FLAIR lesion volume but not CE tumor volume was significantly higher in patients demonstrating volumetric changes in comparison to those who did not (pre-op: *p* = * 0.009 *p* = 0.629; post-op: *p* = * 0.007 *p* = 0.258). Accordingly, RANO *resect* classes were different between both groups, with patients displaying volumetric changes being more frequently allocated to RANO classes two and three (*p* = * 0.033).

### 3.3. Predictors of Outcome

**Figure 2:**
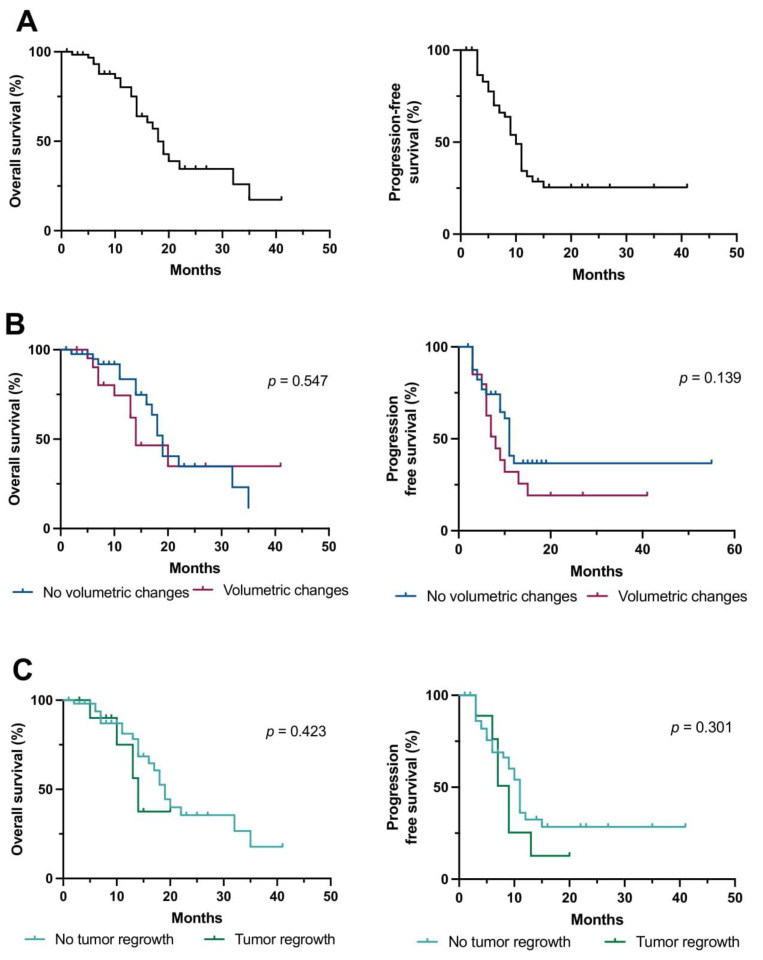
Survival after microsurgical tumor resection depending on volumetric changes between surgery and initiation of adjuvant therapy. (**A**–**C**) Kaplan–Meier estimates of overall and progression-free survival in the entire cohort (**A**), comparing patients with (red curve) or without (blue curve) volumetric changes of CE and non-CE lesions between immediate postoperative MRI and imaging obtained before radiotherapy (**B**), and comparing patients with imaging findings displaying tumor regrowth (green curve) and those with without (light blue curve). Tick marks indicate censored patients. Abbreviations: CE—contrast-enhancing; non-CE—non-contrast-enhancing.

At the time of the database closure, 38/64 patients (60%) were alive (23 of those without first progression), including five of sixty-four patients (8%) lost to follow-up (not seen > 12 months), and 26/64 patients (41%) had died. Across the entire cohort, the median overall survival was 18 months (range: 2–35 months) and the median time to radiographic progression after first-line therapy was 10 months (range: 0–15 months) at a median follow-up of 11 months (range: 1–41 months), matching recent outcome data for patients with IDHwt glioblastoma (Figure 2A) [3,17,18]. As expected, MGMT promotor methylation was associated with better overall survival in the entire cohort (17 versus 32 months; *p* = * 0.019). Furthermore, we reassessed the established RANO *resect* classes, also on pre-RT MRI (‘RANO *resect* RT’). Interestingly, a class change of ≥1 between RANO *resect* and ‘RANO *resect* RT’ was associated with lower OS in the entire cohort (14 versus 19 months, * *p* = 0.033).

Next, we aimed to analyze whether volumetric changes in tumor remnants or patients displaying tumor regrowth were associated with less favorable survival. Here, OS and PFS were not significantly different in patients with or without volumetric changes of tumor remnants (OS: 14 versus 19 months, *p* = 0.547; PFS: 8 versus 11 months, *p* = 0.139; Figure 2B). This held true when only comparing patients treated with radiochemotherapy (*n* = 54; *p* = 0.368 and *p* = 0.212, respectively). Moreover, there was no association with outcome in patients with imaging findings suggestive for tumor regrowth in the entire patient cohort (OS: *p* = 0.423; PFS: *p* = 0.301; Figure 2C), and in patients treated with radiochemotherapy (*n* = 54; OS: *p* = 0.404; PFS: *p* = 0.182). No other statistically significant predictors of outcome (e.g., KPS, TERT status) other than age ≥ 65 years were identified on univariate analysis.

## 4. Discussion

The frequency and relevance of early MRI changes in glioblastoma patients defined per WHO 2021 classification is so far unclear. Based on a large cohort of 64 IDH-wildtype glioblastoma patients in which immediate postoperative MRI as well as pre-radiotherapy MRI was available, we elaborated on the prognostic role of these early MRI changes.

In the present study, we found a substantial progression of CE and non-CE volumes in about every third patient during the interval between surgery and radiotherapy. Here, true tumor progression was either due to new contrast-enhancement after supramarginal resection, the growth of residual tumor remnants or BBB breakdown translating into new contrast-enhancement in formerly non-CE portions. This is in line with previous reports highlighting that any residual CE tumor is associated with decreased progression-free (and also overall) survival [19,20]. Moreover, our findings may argue for the surgical targeting of not only the CE but also the non-CE volume, as this appears to represent a source of highly active tumor tissue with the potential for rapid progression [3,21]. On a cautionary note, a multidisciplinary in-depth MRI review showed that an increase in CE or non-CE signals on MRI was also often due to non-malignant causes such as ischemia or other unspecific signals (potentially derived from the surgical manipulation). Accordingly, a high level of suspicion is warranted when evaluating MRI after 72 h following surgery for the extent of resection, and the RANO guidelines recommending early postoperative imaging should therefore be followed. Diffusion weighted sequences help to differentiate ischemic lesions [22]. Notably, MRI prior to radiotherapy for the purpose of target volume delineation according to the current ESTRO-ACROP guidelines seems appropriate as we observed selected cases with new distant CE foci, which might otherwise not have been covered by the standard radiation margin [10]. Apart from postoperative structural MRI imaging, metabolic imaging modalities such as [^18^F]fluorethyltyrosine positron emission tomography ([^18^F]FET-PET) represent a promising tool to evaluate postoperative tumor remnants after microsurgical resection, and may be helpful for adjuvant radiotherapy planning [23,24].

When assessing outcome differences between patients with and without early tumor progression, we failed to find any distinct survival association with such MRI changes. Importantly, our sample size was limited, and our results could have been confounded due to the retrospective nature of the study as well as the different adjuvant therapy regimes applied. Whereas some studies have also been unable to find such prognostic differences [25], others were more in support of such an association [26,27]. However, only two studies controlled for IDH status as required to the diagnosis of ‘glioblastoma WHO grade 4’ per the current WHO 2021 classification [8,9]. In our study, we ruled out that our results have been confounded by such molecular, but also by clinical radiographic markers. Most remarkably, all patients included in the outcome analysis were homogenously treated with radiochemotherapy per current standard of care [2]. Only TERT mutations have been more frequently observed among patients with early progression; however, prior reports found no prognostic relevance of the TERT promotor status in glioblastoma patients when other diagnostic hallmarks have been fulfilled (as in our series) [28]. Moreover, patients over 65 years were associated with unfavorable outcomes; however, hypofractionated radiotherapy more frequently used in elderly patients could have been a confounding factor in this context.

It is tempting to speculate whether we failed to observe a survival difference in patients with true early progression since the target volume for radiation was adjusted accordingly in these patients using the second, pre-radiotherapy scan. Further prospective studies are warranted in this regard. Additionally, very substantial volumetric changes translating into a change of the RANO class for the extent of resection may, indeed, harbor some prognostic relevance.

On a cautionary note, the sample size was small, which limited the power of our study, and potential bias introduced by our retrospective study cohort has to be taken into account. Prospective, preferably large-scale multicenter studies will need to address whether the target delineation based upon pre-radiotherapy MRI may come with an increased disease control rate and comparable low toxicities.

## 5. Conclusions

In conclusion, contrast-enhancing and non-contrast-enhancing progressions of tumor portions between MRI after surgery and prior to radiotherapy in glioblastoma patients are commonly encountered. However, in-depth analysis revealed not only tumor regrowth but also a breakdown of the blood–brain barrier in previously non-enhancing compartments, with frequent local (clinically non-apparent) postoperative ischemia or unspecific signals responsible for said changes. Immediate postoperative MRI seems more reliable for stratification for the extent of resection after microsurgical tumor removal, while additional imaging obtained before radiotherapy might be helpful for better tumor volume delineation and therapy planning, respectively.

## Figures and Tables

**Table 1:** Baseline characteristics for patients with glioblastoma, IDH-wildtype, CNS WHO grade 4.

Characteristics	Categories	Patients without Volumetric Changes	Patients with (+) Volumetric Changes	Total	*p*-Value
**Overall, *n* (%)**		42 (66%)	22 (34%)	64 (100%)	
**Age, years (%)**	Mean	61.6	57.2	60.1	0.123
	18–50	8 (19%)	4 (18%)	12 (19%)	
	51–65	16 (38%)	13 (59%)	29 (45%)	
	>65	18 (43%)	5 (23%)	23 (36%)	
**Sex, *n* (%)**	Female	11 (26%)	10 (45%)	21 (33%)	0.163
	Male	31 (74%)	12 (55%)	43 (67%)	
**Clinical performance**	Pre-op KPS, median (range)	80 (60–100%)	90 (20–100%)	85 (20–100%)	0.133
	Post-op KPS, median (range)	80 (50–100%)	90 (50–90%)	80 (50–100%)	0.394
	New postoperative deficit, *n* (%)	3 (7%)	5 (23%)	8 (13%)	0.111
**MGMT promotor, *n* (%)**	Methylated	12 (29%)	10 (45%)	22 (34%)	0.268
	Non-methylated	30 (71%)	12 (55%)	42 (66%)	
**TERT promotor, *n* (%)**	Wildtype	10 (24%)	0 (0%)	10 (16%)	* 0.012
	Mutated	32 (76%)	22 (100%)	54 (84%)	
**Tumor localization, *n* (%)**	(Sub)-cortical	38 (90%)	18 (82%)	56 (88%)	0.430
	Multifocal	1 (2%)	4 (18%)	5 (8%)	
	Deep-seated	2 (5%)	0 (0%)	2 (3%)	
	Cerebellar	1 (2%)	0 (0%)	1 (2%)	
	Dominant hemisphere	20 (48%)	10 (45%)	30 (47%)	0.999
**Tumor volumes,** **cm^3^; mean ± SEM**	Pre-op CE	30.9 ± 3.8	34.1 ± 5.3	32.0 ± 3.1	0.624
	Pre-op non-CE	47.7 ± 5.4	66.0 ± 6.2	53.9 ± 4.2	* 0.009
	Post-op CE	0.5 ± 0.2	2.3 ± 1.2	1.1 ± 0.4	0.258
	Post-op non-CE	2.2 ± 0.6	12.2 ± 4.1	5.7 ± 1.6	* 0.007
	Pre-RT CE	0.5 ± 0.2	6.2 ± 2.5	2.4 ± 0.9	* 0.001
	Pre-RT non-CE	2.2 ± 0.6	19.8 ± 5.4	8.3 ± 2.1	* 0.001
**RANO categories of EOR in glioblastoma, *n* (%)**	RANO class 1	28 (67%)	8 (36%)	36 (56%)	* 0.033
	RANO class 2	6 (14%)	9 (41%)	15 (23%)	
	RANO class 3	8 (19%)	5 (23%)	13 (20%)	
**Adjuvant therapy**	EORTC-26981/22981	28 (67%)	19 (86%)	47 (73%)	0.137
	RT alone	9 (21%)	1 (5%)	10 (16%)	
	EORTC-26981/22981 + study drug	3 (7%)	1 (5%)	4 (6%)	
	CeTeG	2 (5%)	1 (5%)	3 (5%)	

Characteristics are given for all patients with glioblastoma, IDHwt, CNS WHO grade 4 (*n* = 64), patients without (*n* = 42) and with (*n* = 22) volumetric changes on MRIs between surgery and radiotherapy. Significant differences are indicated by * *p* ≤ 0.05. Abbreviations: (+)—positive volume changes; CE—contrast-enhancing; CCNU—lomustine; CeTeG—TMZ + CCNU/RT → TMZ + CCNU; EOR—extent of resection; EORTC-26981/22981—TMZ/RT → TMZ; KPS—Karnofsky Performance Status; MGMT—O6-methylguanine-DNA-methlytransferase; non-CE—non-contrast-enhancing; Post-op—postoperative; Pre-op—pre-operative; Pre-RT—pre-radio(chemo)therapy; RT—radiotherapy; TERT—telomerase reverse transcriptase; TMZ—temozolomide.

## Data Availability

The data presented in this study are available on request from the corresponding authors. The data are not publicly available due to the guidelines of the Institutional Review Board of the Ludwig-Maximilians-University in Munich.

## Supplementary Materials

The following supporting information can be downloaded at: https://www.mdpi.com/article/10.3390/cancers15061745/s1, Figure S1: Flow diagram of patient selection.
