# Supplementary material for: Frequency and Prognostic Relevance of Volumetric MRI Changes in Contrast- and Non-Contrast-Enhancing Tumor Compartments between Surgery and Radiotherapy of IDHwt Glioblastoma
Source: Cancers (Basel). 2023 Mar 14;15(6):1745. doi: 10.3390/cancers15061745 (PMC10046652; doi:10.3390/cancers15061745)
Supplement: Supplementary file 1 [file cancers-15-01745-s001.zip › cancers-2168082-supplementary.pdf]

cancers MDPI

Supplementary Materials

Frequency and Prognostic Relevance of Volumetric MRI Changes in Contrast- and Non-Contrast-Enhancing Tumor Compartments between Surgery and Radiotherapy of IDHwt Glioblastoma

Nico Teske, Nina C. Teske, Maximilian Niyazi, Claus Belka, Niklas Thon, Joerg-Christian Tonn, Robert Forbrig and Philipp Karschnia

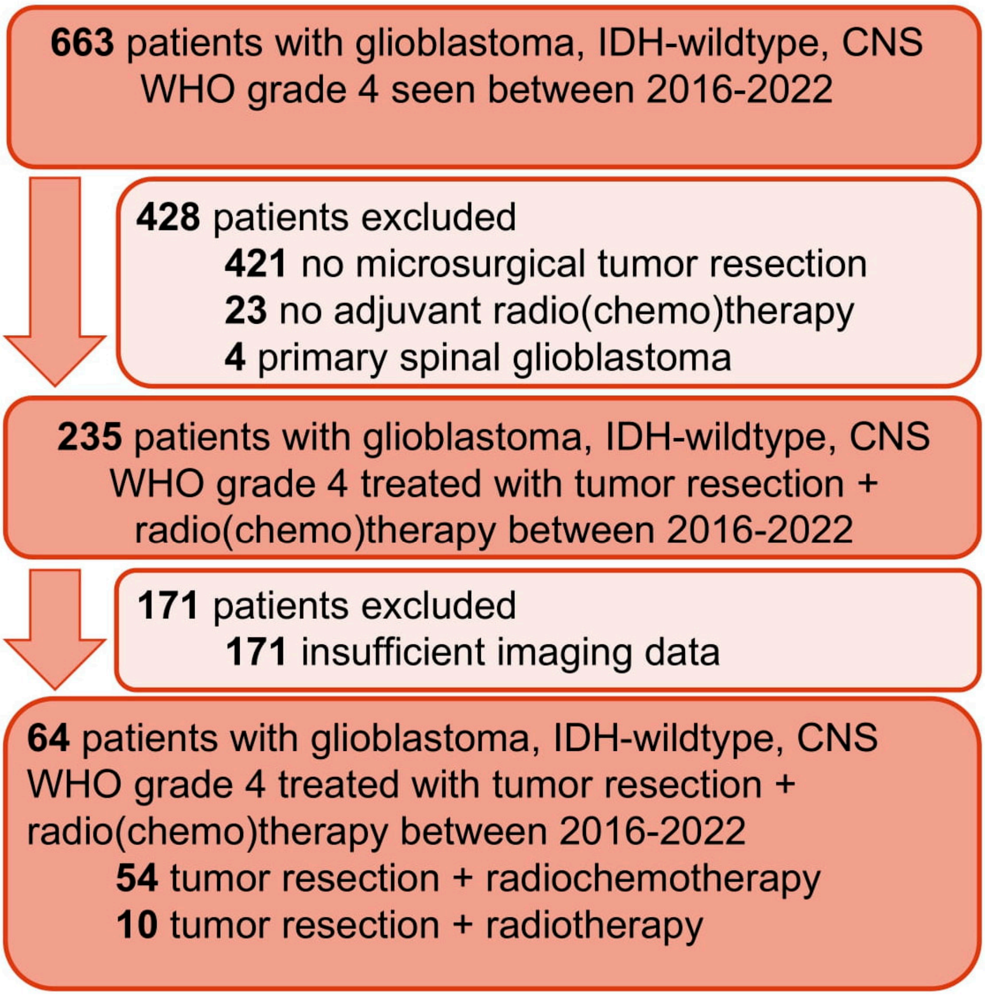

Figure S1. Flow diagram of patient selection. Schematic representation reporting the selection of the entire patient cohort of newly diagnosed glioblastoma patients undergoing microsurgical tumor resection at the Centre for Neuro-Oncology at the Ludwig-Maximilians-University School of Medicine (n = 64).
